# Association between post-traumatic stress disorder symptoms and bone fractures after the Great East Japan Earthquake in older adults: a prospective cohort study from the Fukushima Health Management Survey

**DOI:** 10.1186/s12877-020-01934-9

**Published:** 2021-01-07

**Authors:** Fumikazu Hayashi, Tetsuya Ohira, Hironori Nakano, Masanori Nagao, Kanako Okazaki, Mayumi Harigane, Seiji Yasumura, Masaharu Maeda, Atsushi Takahashi, Hirooki Yabe, Yuriko Suzuki, Kenji Kamiya, the Fukushima Health Management Survey Group

**Affiliations:** 1 Department, of Epidemiology, School of Medicine, Fukushima Medical University, 1 Hikarigaoka, Fukushima-city, Fukushima, 960-1295 Japan; 2 Radiation Medical Science Center for the Fukushima Health Management Survey, Fukushima Medical University, 1 Hikarigaoka, Fukushima-city, Fukushima, 960-1295 Japan; 3 Department of Public Health, School of Medicine, Fukushima Medical University, 1 Hikarigaoka, Fukushima-city, Fukushima, 960-1295 Japan; 4 Department of Disaster Psychiatry, School of Medicine, Fukushima Medical University, 1 Hikarigaoka, Fukushima-city, Fukushima 960-1295 Japan; 5 Department of Gastroenterology, School of Medicine, Fukushima Medical University, 1 Hikarigaoka, Fukushima-city, Fukushima 960-1295 Japan; 6 Department of Neuropsychiatry, School of Medicine, Fukushima Medical University, 1 Hikarigaoka, Fukushima-city, Fukushima 960-1295 Japan; 7 Department of Mental Health Policy, National Institute of Mental Health, National Center of Neurology and Psychiatry, 4-1-1 Ogawa-Higashi, Kodaira, Tokyo 187-8553 Japan

**Keywords:** Fractures, Mental health, Aged, Disaster victims, Fukushima nuclear accident

## Abstract

**Background:**

It has been reported that psychological stress affects bone metabolism and increases the risk of fracture. However, the relationship between bone fractures and post-traumatic stress disorder (PTSD) is unclear. This study aimed to evaluate the effects of disaster-induced PTSD symptoms on fracture risk in older adults.

**Methods:**

This study evaluated responses from 17,474 individuals aged ≥ 65 years without a history of fractures during the Great East Japan Earthquake who answered the Mental Health and Lifestyle Survey component of the Fukushima Health Management Survey conducted in 2011. The obtained data could determine the presence or absence of fractures until 2016. Age, sex, physical factors, social factors, psychological factors, and lifestyle factors were subsequently analyzed. Survival analysis was then performed to determine the relationship between the fractures and each factor. Thereafter, univariate and multivariate Cox proportional hazard models were constructed to identify fracture risk factors.

**Results:**

In total, 2,097 (12.0%) fractures were observed throughout the follow-up period. Accordingly, univariate and multivariate Cox proportional hazard models showed that PTSD symptoms (total PTSD checklists scoring ≥ 44) [hazard ratio (HR): 1.26; 95% confidence interval (CI): 1.10–1.44; *P* = 0.001], history of cancer (HR: 1.49; 95% CI: 1.24–1.79; *P* < 0.001), history of stroke (HR: 1.25; 95% CI: 1.03–1.52; *P* = 0.023), history of heart disease (HR: 1.30; 95% CI: 1.13–1.50; *P* < 0.001), history of diabetes (HR: 1.23; 95% CI: 1.09–1.39; *P* < 0.001), current smoking (HR: 1.29; 95% CI: 1.02–1.63; *P* = 0.036), and high dissatisfaction with sleep or no sleep at all (HR: 1.33; 95% CI: 1.02–1.74; *P* = 0.035) promoted a significant increase in fracture risk independent of age and sex.

**Conclusions:**

The present study indicates that disaster-induced PTSD symptoms and insomnia contribute to increased fracture risk among older adults residing in evacuation areas within the Fukushima Prefecture.

## Background

The Great East Japan Earthquake occurred with its epicenter in the sea floor, 130 km off the southeast Oshika Peninsula, Miyagi Prefecture, on March 11, 2011 [1], subsequently triggering the Fukushima Daiichi Nuclear Power Station (FDNPS) accident in Fukushima Prefecture. Accordingly, the number of adult inhabitants of the evacuation zone with post-traumatic stress disorder (PTSD) checklist (PCL) scores that were above the cut-off value was comparable to that of workers affected by the 9/11 World Trade Center attack [2, 3]. A survey of 240 evacuees in the evacuation area of Hirono Town, Fukushima Prefecture, revealed that 66.8 and 53.5% of evacuees had reported clinically relevant symptoms of depression and PTSD, respectively [4]. Thus, residents of evacuation areas, such as those in Fukushima Prefecture, could have presented with PTSD symptoms caused by disaster-related events.

A study recently reported a possible relationship between increased fracture risk and PTSD [5]. Therefore, residents in Fukushima evacuation areas who presented with PTSD symptoms could have also been at high risk for fractures. The results of the 2016 Basic Survey on National Life published by the Japanese Ministry of Health, Labor, and Welfare revealed that 12.1% of the 100,000 individuals requiring care had been certified as requiring support or nursing care because of falls or broken bones—major factors equivalent to arthritis or infirmity caused by aging [6]. Moreover, Tanji et al. reported that those with higher psychological distress after an earthquake had a higher risk for requiring nursing care than those with lower psychological distress [7]. Accordingly, the associated higher risk for increased fractures among residents in evacuation areas within Fukushima Prefecture presenting with PTSD symptoms could affect their healthy life expectancy and quality of life (QOL). In particular, the increased fracture risk among older adults could contribute to an increase in the number of those requiring support or nursing care. However, no study has examined the relationship between earthquake-induced PTSD symptoms and fractures in older adult residents of evacuation areas within Fukushima Prefecture. Therefore, investigating the association between PTSD symptoms and fractures among such residents is imperative to maintain and improve their healthy life expectancy and QOL.

We used data from the Fukushima Health Management Survey to investigate the relationship between bone fractures and PTSD symptoms after the Great East Japan Earthquake in older adults.

## Methods

### Study group

The Fukushima Health Management Survey was initiated in the Fukushima Prefecture on January 18, 2012 to investigate and observe the health status of evacuees [8]. Individuals who completed the Fukushima Health Management Survey, including the Mental Health and Lifestyle Survey component, were among those residing in any of the 13 municipalities (all areas within Hirono-machi, Naraha-machi, Tomioka-machi, Kawauchi-mura, Okuma-machi, Futaba-machi, Namie-machi, Katsurao-mura, and Iitate-mura, as well as parts of Tamura city, Minamisoma city, Kawamata town, and Date city) who had to be evacuated because of the Great East Japan Earthquake (registered residents) [9].

A total of 180,604 individuals aged ≥ 15 years (born before April 1, 1995) were eligible for the 2011 edition of the registered questionnaire [10]. Valid responses with a response rate of 40.7% were obtained from 73,431 individuals (mean age: 56.4 years). After excluding 46,365 individuals aged < 65 years, 1,220 individuals with an unknown fracture history, and 3,933 individuals who already had a history of fractures in 2011, a total of 21,913 individuals aged ≥ 65 years (10,271 men; 11,642 women; mean age: 75.0 ± 6.9 years) comprised the sample for the present study.

**Fig. 1:**
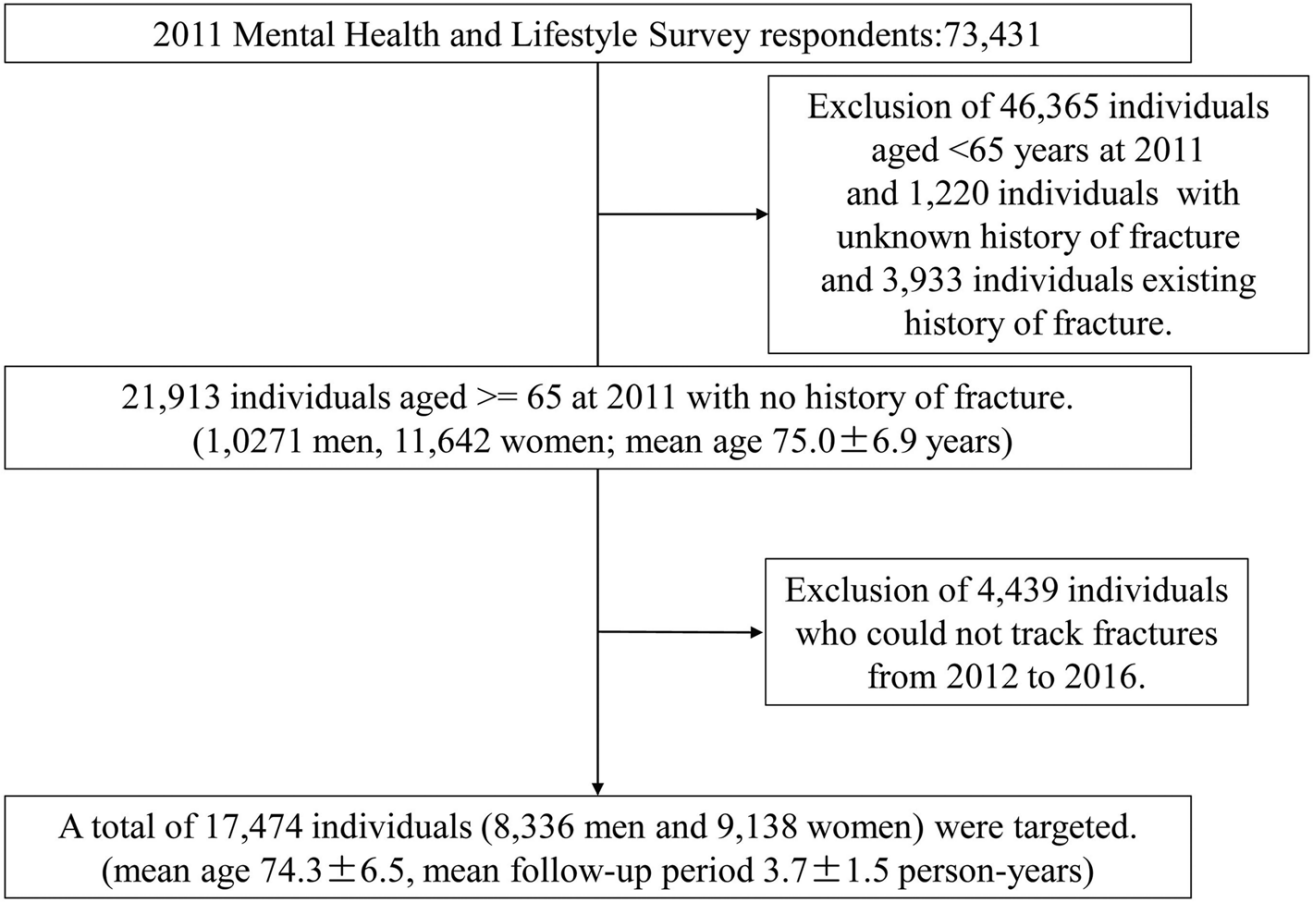
Selection of study participants

Incidences of fractures between 2012 and 2016 were determined using the questionnaire on fractures. Accordingly, 4,439 individuals were further excluded due to missing fracture data from 2012 to 2016 given that they had never responded to a questionnaire after 2011. Ultimately, 17,474 patients (9,138 women; 8,336 men; mean age, 74.3 ± 6.5 years; mean follow-up duration, 3.7 ± 1.5 person-years) were targeted (Fig. 1).

Data regarding the age, sex, physical factors (history of fractures, cancer, stroke, heart disease, diabetes, dyslipidemia, hepatic disorder, high blood pressure, and thyroid disease), social factors [experience of the earthquake, tsunami, and nuclear power plant accident (heard the explosion); need for assistance; change in employment status; and change in residence], psychological factors (history of mental illness and PCL), and lifestyle factors (history of smoking and drinking, sleep satisfaction levels, and exercise habits) obtained from the self-administered questionnaire items used in the 2011 Mental Health and Lifestyle Survey were herein analyzed.

### Fracture determination

In the Mental Health and Lifestyle Survey, questions on the presence or absence of fractures differed depending on the year. Thus, fracture incidences were determined by combining these questions.

The presence or absence of fractures in 2011 and 2012 was confirmed by responding to a question on “A history of fractures after age 50.” In 2013, in addition to the aforementioned question, a combination of answers on whether “a fracture was diagnosed by a physician within the past year” was used to determine the presence or absence of fractures. In 2014 and 2015, the presence or absence of fractures was determined based on the answer to “fractures within 1 year” alone. In 2016, the incidence of fractures was determined by a question on “History of fractures after the age of 50.”

### Definition of estimated fracture occurrence date and calculation of follow-up period

The questionnaire used herein could not determine the date on which the fracture occurred. Therefore, this study estimated fracture occurrence dates by identifying the midpoint between the date on which the questionnaire revealed that no fracture occurred during the year and the date on which the questionnaire revealed that a fracture occurred during that year or 6 months before the date on which the questionnaire revealed that a fracture occurred during that year.

Furthermore, a number of participants also had trouble completing the questionnaire, particularly with regard to information on the month and date of completion, rendering it impossible to calculate the follow-up period. The questionnaire was distributed via mail in the month of February of the survey year (e.g., for 2011, the questionnaire was distributed in February 2012). A breakdown of the months during which the questionnaires had been filled out showed that approximately 77%–87% were filled out in February for each year. Therefore, for missing information of the month in which they responded, we assumed that the individuals responded in the month of February for that year. Moreover, when information regarding the date on which the questionnaire was answered was missing, we assumed that they responded to the questionnaire on the 15^th^ of that month.

### Evaluation of post-traumatic stress disorder symptoms

The presence or absence of PTSD symptoms was evaluated using PCL [11], a self-administered questionnaire that obtained information on the symptoms of PTSD according to the diagnostic criteria in the Diagnostic and Statistical Manual of Mental Disorders-IV. The reliability, validity, and diagnostic efficiency of the Japanese version of the PCL, which was used to determine PTSD symptoms among residents who experienced the Fukushima nuclear accident, have been previously established [12]. The respondents were asked to answer a total of 17 questions using a five-point Likert scale. Accordingly, individuals with higher total scores were strongly suspected to have PTSD. Moreover, a previous study determined that a total PCL score of ≥ 44 points was the cut-off for suspecting the presence of PTSD symptoms [13]. In this study, the total PCL score was calculated only for individuals who answered ≥ 16 questions. When only 16 questions were answered, the average score for the 16 questions was assigned to the missing items to calculate the total score. Participants with total PCL scores ≥ 44 were defined as those “with PTSD symptoms” and examined.

### History of disease

Residents were asked whether they had a history of cancer, stroke, heart disease, hypertension, diabetes, dyslipidemia, hepatic disorder, thyroid disease, or mental illness.

### Lifestyle

The questionnaire section on smoking habits had three choices: never smoked, former smoker, or current smoker. The section on drinking habits also had three choices: never drinks or rarely drinks (less than once a month), former drinker, or current drinker (more than once a month). The question on sleep satisfaction had four choices: satisfied with sleep, slightly unsatisfied with sleep, quite unsatisfied with sleep, and very dissatisfied with sleep or does not sleep at all [9]. Furthermore, the question on exercise habits had four choices: almost daily, two to four times a week, approximately once a week, or almost never.

### Experience of the Great East Japan Earthquake

In the questionnaire on the experience of the Great East Japan Earthquake, individuals responded to whether they had experienced the earthquake, tsunami, and nuclear power plant accident (heard the explosion).

### Need for assistance

In the question on need for assistance, individuals responded to whether they could eat, change clothes, use restrooms, and shop independently. Individuals who answered that assistance was required for any of the aforementioned four items were defined as those requiring assistance.

### Changes in employment status

Residents could respond with “changed” or “unchanged” with regard to change in employment status (job change or unemployment) following the earthquake and accident [9].

### Changes in housing and evacuation

Based on the question on change in residence [9], residents who lived in temporary or evacuation shelters immediately after the earthquake were defined as those who changed their residence.

Furthermore, residents of Tamura city, Minamisoma city, Date city, and Kawamata town who did not reside in a temporary or evacuation center in 2011 were defined as those who were not evacuated. Others were defined as those who were evacuated.

### Statistical analysis methods

All statistical analyses were performed using SAS 9.4 (SAS Institute Inc., Cary, NC, USA). The Kaplan–Meier method and log-rank test were used to compare difference in the incidence of fractures based on questionnaire responses. Moreover, univariate and multivariate Cox proportional hazards models were used to obtain crude and adjusted hazard ratios (HRs) and 95% confidence intervals (CIs) for the association between each factor and fractures. Furthermore, multivariate Cox proportional hazards models for men and women were established to determine differences according to sex.

In this study, it is necessary to consider the possibility that differences in questioning for fractures in each year and incomplete tracking may introduce selection and information bias. Therefore, as a sensitivity analysis, we confirmed the robustness of the results by performing a Cox regression analysis limited to individuals without missing data in the fracture questionnaire for all years.

All data are presented as number of individuals (n), mean, standard deviation, median, 25^th^ percentile, 75^th^ percentile, or percentages. *P* < 0.05 indicated statistical significance.

## Results

### Participant characteristics

Table 1 summarizes the participants’ characteristics. A total of 2,097 (12.0%) participants experienced a fracture during the follow-up period, with an incidence rate of 0.032/year.

**Table 1:** The association between fracture and mental health and lifestyle survey items

Factor	Classification	All participants (*n* = 17,474)		Nonfracture group (*n* = 15,377)		Fracture group (*n* = 2,097)		*P* value
		Mean	SD	Mean	SD	Mean	SD	
Age	Years	74.3	6.5	74.2	6.5	75.5	6.7	
Follow–up period	Person–years	3.7	1.5	3.9	1.4	2.0	1.4	
		n	%	n	%	n	%	
Sex	Men	8,336	47.7	7,550	49.1	786	37.5	< 0.001
	Women	9,138	52.3	7,827	50.9	1,311	62.5	
PTSD symptoms	No	11,688	74.5	10,451	75.3	1,237	67.9	< 0.001
	Yes	4,009	25.5	3,423	24.7	586	32.1	
Experience of evacuation	No	9,382	54.0	8,298	54.0	1,084	51.7	0.228
	Yes	8,092	46.0	7,079	46.0	1,013	48.3	
Experience of earthquake	No	1,238	7.1	1,072	7.0	166	7.9	0.017
	Yes	16,236	92.9	14,305	93.0	1,931	92.1	
Experience of tsunami	No	13,452	77.0	11,821	76.9	1,631	77.8	0.309
	Yes	4,022	23.0	3,556	23.1	466	22.2	
Experience of nuclear accident (explosion heard)	No	6,941	39.7	6,088	39.6	853	40.7	0.169
	Yes	10,533	60.3	9,289	60.4	1,44	59.3	
History of mental illness	No	16,066	94.8	14,170	95.0	1,896	93.4	< 0.001
	Yes	875	5.2	741	5.0	134	6.6	
Need for assistance	No	15,541	91.3	13,745	91.7	1,796	88.2	< 0.001
	Yes	1,489	8.7	1,249	8.3	240	11.8	
History of cancer	No	15,243	91.8	13,450	92.1	1,793	90.0	< 0.001
	Yes	1,356	8.2	1,156	7.9	200	10.0	
History of stroke	No	15,055	89.9	13,287	90.3	1,768	87.4	< 0.001
	Yes	1,688	10.1	1,434	9.7	254	12.6	
History of heart disease	No	13,613	81.0	12,068	81.7	1,545	76.6	< 0.001
	Yes	3,185	19.0	2,712	18.3	473	23.4	
History of diabetes mellitus	No	10,969	65.9	9,695	66.2	1,274	63.4	0.004
	Yes	5,676	34.1	4,942	33.8	734	36.6	
History of dyslipidemia	No	8,316	49.6	7,336	49.7	980	48.6	0.723
	Yes	8,459	50.4	7,421	50.3	1,038	51.4	
History of hepatic disorder	No	16,130	96.7	14,200	96.8	1,930	96.2	0.069
	Yes	545	3.3	469	3.2	76	3.8	
History of hypertension	No	4,986	29.1	4,405	29.3	581	28.2	0.182
	Yes	12,123	70.9	10,644	70.7	1,479	71.8	
History of thyroid disease	No	16,611	97.1	14,632	97.1	1,979	96.8	0.333
	Yes	497	2.9	431	2.9	66	3.2	
Smoking habit	Never smoked	10,187	61.3	8,866	60.5	1,321	67.3	< 0.001
	Former smoker	4,782	28.8	4,317	29.5	465	23.7	
	Current smoker	1,644	9.9	1,468	10.0	176	9.0	
Drinking habit	Never drinks or rarely drinks (less than once a month)	9,307	55.2	8,121	54.6	1,186	59.3	< 0.001
	Former drinker	958	5.7	847	5.7	111	5.6	
	Current drinker (more than once a month)	6,596	39.1	5,893	39.7	703	35.2	
Level of sleep satisfaction	Satisfied with sleep	5,309	41.9	4,738	42.6	571	36.9	< 0.001
	Slightly unsatisfied with sleep	5,122	40.4	4,488	40.4	634	41.0	
	Quite unsatisfied with sleep	1,650	13.0	1,408	12.7	242	15.6	
	Very dissatisfied with sleep or does not sleep at all	583	4.6	483	4.3	100	6.5	
Exercise habit	Almost daily	4,303	25.9	3,829	26.2	474	23.9	0.003
	2 to 4 times a week	5,231	31.5	4,620	31.6	611	30.8	
	Approximately once a week	2,505	15.1	2,193	15.0	312	15.7	
	Almost never	4,586	27.6	4,000	27.3	586	29.6	
Job change	No	8,242	54.5	7,297	54.6	945	53.7	0.884
	Yes	6,874	45.5	6,058	45.4	816	46.3	
Loss of job	No	15,132	86.6	13,307	86.5	1,825	87.0	0.145
	Yes	2,342	13.4	2,070	13.5	272	13.0	
Residential changes	No	10,355	62.1	9,135	62.2	1,220	61.4	0.738
	Yes	6,324	37.9	5,557	37.8	767	38.6	

Data are presented as a number with a percentage or a mean with standard deviation

The interval scale between the bone fracture and no bone fracture group groups was tested using the log–rank test

*SD* standard deviation, *PTSD* post–traumatic stress disorder

*p* < 0.05 was considered statistically significant

### Survival analysis results

The relationship between each factor and the incidence of fractures was examined among participants divided into the fracture and nonfracture groups (Table 1). Accordingly, survival analysis results found significant differences in fracture incidence among older adults according to sex (*P* < 0.001), PTSD symptoms (*P* < 0.001), experience of earthquake (*P* = 0.017), history of mental illness (*P* < 0.001), need for assistance (*P* < 0.001), history of cancer (*P* < 0.001), history of stroke (*P* < 0.001), history of heart disease (*P* < 0.001), history of diabetes (*P* = 0.004), smoking habits (*P* < 0.001), drinking habits (*P* < 0.001), sleep satisfaction (*P* < 0.001), and exercise habits (*P* = 0.003).

### Univariate and multivariate Cox proportional hazards models

Univariate and multivariate Cox proportional hazards models were established using factors that were determined to be significant during survival analysis to identify the association between psychological indicators and fracture frequency among older adults (Table 2). Accordingly, the multivariate and univariate Cox proportional hazards analyses showed that PTSD symptoms (HR: 1.26; 95% CI: 1.10–1.44; *P* = 0.001), history of cancer (HR: 1.49; 95% CI: 1.24–1.79; *P* < 0.001), history of stroke (HR: 1.25; 95% CI: 1.03–1.52; *P* = 0.023), history of heart disease (HR: 1.30; 95% CI: 1.13–1.50; *P* < 0.001), history of diabetes (HR: 1.23; 95% CI: 1.09–1.39; *P* < 0.001), current smoking(HR: 1.29; 95% CI: 1.02–1.63; *P* = 0.036), and high dissatisfaction with sleep or no sleep at all (HR: 1.33; 95% CI: 1.02–1.74; *P* = 0.035) significantly increased fracture risk, independent of age and sex.

**Table 2:** The results of univariate and multivariate Cox proportional hazard models

Factors	Classification	Nonfracture group (*n* = 15,377)	Fracture group (*n* = 2,097)	Crude HR (95% CI)	*P* value	Adjusted HR (95% CI) ^a^ (*n* = 10,032)	*P* value
Age	Continuous			1.04 (1.04–1.05)	< 0.001	1.04 (1.03–1.05)	< 0.001
Sex	Men	7,550	786	Ref		Ref	
	Women	7,827	1,311	1.59 (1.45–1.73)	< 0.001	1.85 (1.55–2.20)	< 0.001
PTSD symptoms	No	10,451	1,237	Ref		Ref	
	Yes	3,423	586	1.43 (1.30–1.58)	< 0.001	1.26 (1.10–1.44)	0.001
Experience of earthquake	No	1,072	166	Ref		Ref	
	Yes	14,305	1,931	0.82 (0.70–0.97)	0.017	0.92 (0.69–1.20)	0.531
History of mental illness	No	14,170	1,896	Ref		Ref	
	Yes	741	134	1.45 (1.21–1.72)	< 0.001	0.98 (0.76–1.27)	0.869
Need for assistance	No	13,745	1,796	Ref		Ref	
	Yes	1,249	240	1.85 (1.62–2.12)	< 0.001	1.14 (0.92–1.41)	0.240
History of cancer	No	13,745	1,796	Ref		Ref	
	Yes	1,249	240	1.31 (1.13–1.51)	< 0.001	1.49 (1.24–1.79)	< 0.001
History of stroke	No	13,287	1,768	Ref		Ref	
	Yes	1,434	254	1.41 (1.24–1.61)	< 0.001	1.25 (1.03–1.52)	0.023
History of heart disease	No	12,068	1,545	Ref		Ref	
	Yes	2,712	473	1.37 (1.23–1.51)	< 0.001	1.30 (1.13–1.50)	< 0.001
History of diabetes mellitus	No	9,695	1,274	Ref		Ref	
	Yes	4,942	734	1.14 (1.04–1.25)	0.004	1.23 (1.09–1.39)	< 0.001
Smoking habit	Never smoked	8,866	1,321	Ref		Ref	(trend *p* = 0.057)
	Former smoker	4,317	465	0.73 (0.66–0.81)	< 0.001	1.03 (0.86–1.24)	0.739
	Current smoker	1,468	176	0.83 (0.71–0.97)	0.022	1.29 (1.02–1.63)	0.036
Drinking habit	Never drinks or rarely drinks (less than once a month)	8,121	1,186	Ref		Ref	(trend *p* = 0.134)
	Former drinker	847	111	0.95 (0.78–1.15)	0.595	1.22 (0.92–1.63)	0.171
	Current drinker (more than once a month)	5,893	703	0.79 (0.72–0.87)	< 0.001	1.13 (0.98–1.31)	0.105
Level of sleep satisfaction	Satisfied with sleep	4,738	571	Ref		Ref	(trend *p* = 0.142)
	Slightly unsatisfied with sleep	4,488	634	1.15 (1.02–1.28)	0.018	1.04 (0.92–1.19)	0.552
	Quite unsatisfied with sleep	1,408	242	1.40 (1.20–1.62)	< 0.001	1.03 (0.85–1.24)	0.796
	Very dissatisfied with sleep or does not sleep at all	483	100	1.69 (1.37–2.09)	< 0.001	1.33 (1.02–1.74)	0.035
Exercise habit	Almost daily	3,829	474	Ref		Ref	(trend *p* = 0.359)
	2 to 4 times a week	4,620	611	1.06 (0.94–1.20)	0.338	1.00 (0.85–1.17)	0.976
	Approximately once a week	2,193	312	1.16 (1.00–1.33)	0.047	1.08 (0.89–1.30)	0.443
	Almost never	4,000	586	1.24 (1.10–1.40)	< 0.001	1.06 (0.90–1.25)	0.480

^a^Adjusted for age, sex, PCL score, experience of earthquake, history of mental illness, need for assistance, history of cancer, history of stroke, history of heart disease, history of diabetes mellitus, smoking habit, drinking habit, level of sleep satisfaction, and exercise habit. *95% CI* 95% confidence interval, *HR* hazard ratio, *Ref* reference, *PTSD* post–traumatic stress disorder. Cox proportional hazard model; *p* < 0.05 was considered statistically significant

Table 3 presents the results of multivariate Cox proportional hazards analysis according to sex to determine the sex-related differences. Accordingly, PTSD symptoms (HR: 1.39; 95% CI: 1.11–1.74; *P* = 0.004), history of cancer (HR: 1.50; 95% CI: 1.16–1.95; *P* = 0.002), history of diabetes (HR: 1.35; 95% CI: 1.12–1.63; *P* = 0.001), and high dissatisfaction with sleep or no sleep at all (HR: 1.71; 95% CI: 1.12–2.60; *P* = 0.013) had significantly increased fracture risk among older men, independent of age. In contrast, histories of cancer (HR: 1.45; 95% CI: 1.11–1.90; *P* = 0.007) and heart disease (HR: 1.36; 95% CI: 1.13–1.64; *P* = 0.001) significantly increased fracture risk among older women, independent of age. The primary conclusions obtained herein remained largely the same regardless of whether the entry date was supplied.

**Table 3:** The results of multivariate Cox proportional hazard models by sex

Factors	Classification	Men (*n* = 5,185)		Women (*n* = 4,847)	
		Adjusted HR (95% CI)^a^	*P* value	Adjusted HR (95% CI)^b^	*P* value
Age	Continuous	1.03 (1.02–1.05)	< 0.001	1.04 (1.03–1.06)	< 0.001
PTSD symptoms	No	Ref		Ref	
	Yes	1.39 (1.11–1.74)	0.004	1.18 (0.99–1.41)	0.067
Experience of earthquake	No	Ref		Ref	
	Yes	0.86 (0.59–1.25)	0.433	0.92 (0.62–1.38)	0.698
History of mental illness	No	Ref		Ref	
	Yes	0.90 (0.58–1.38)	0.646	1.02 (0.74–1.40)	0.906
Need for assistance	No	Ref		Ref	
	Yes	0.94 (0.61–1.43)	0.765	1.21 (0.94–1.56)	0.140
History of cancer	No	Ref		Ref	
	Yes	1.50 (1.16–1.95)	0.002	1.45 (1.11–1.90)	0.007
History of stroke	No	Ref		Ref	
	Yes	1.29 (0.99–1.69)	0.061	1.18 (0.89–1.57)	0.253
History of heart disease	No	Ref		Ref	
	Yes	1.21 (0.97–1.50)	0.082	1.36 (1.13–1.64)	0.001
History of diabetes mellitus	No	Ref		Ref	
	Yes	1.35 (1.12–1.63)	0.001	1.14 (0.97–1.34)	0.122
Smoking habit	Never smoked	Ref	(trend *p* = 0.200)	Ref	(trend *p* = 0.116)
	Former smoker	1.02 (0.82–1.26)	0.869	1.10 (0.75–1.62)	0.612
	Current smoker	1.22 (0.92–1.62)	0.166	1.46 (0.95–2.28)	0.087
Drinking habit	Never drinks or rarely drinks (less than once a month)	Ref	(trend *p* = 0.425)	Ref	(trend *p* = 0.210)
	Former drinker	1.28 (0.92–1.77)	0.141	0.90 (0.40–2.04)	0.798
	Current drinker (more than once a month)	1.13 (0.90–1.40)	0.293	1.15 (0.93–1.41)	0.193
Level of sleep satisfaction	Satisfied with sleep	Ref	(trend *p* = 0.029)	Ref	(trend *p* = 0.972)
	Slightly unsatisfied with sleep	0.99 (0.80–1.21)	0.986	1.06 (0.90–1.26)	0.493
	Quite unsatisfied with sleep	1.26 (0.94–1.69)	0.126	0.90 (0.70–1.16)	0.409
	Very dissatisfied with sleep or does not sleep at all	1.71 (1.12–2.60)	0.013	1.14 (0.81–1.62)	0.454
Exercise habit	Almost daily	Ref	(trend *p* = 0.819)	Ref	(trend *p* = 0.310)
	2 to 4 times a week	0.92 (0.73–1.17)	0.503	1.07 (0.86–1.33)	0.530
	About once a week	0.99 (0.74–1.33)	0.955	1.17 (0.90–1.51)	0.240
	Almost never	1.01 (0.78–1.29)	0.966	1.12 (0.90–1.40)	0.321

^a,b^Adjusted for age, PCL score, experience of earthquake, history of mental illness, need for assistance, history of cancer, history of stroke, history of heart disease, history of diabetes mellitus, smoking habit, drinking habit, level of sleep satisfaction, and exercise habit. *95% CI* 95% confidence interval, *HR* hazard ratio, *Ref* reference, *PTSD* post–traumatic stress disorder. Cox proportional hazard model; *p* < 0.05 was considered statistically significant

### Sensitivity analysis

In this study, it is important to consider the possibility that differences in questions for fractures in each year and incomplete tracking may introduce selection and information bias. Therefore, as a sensitivity analysis, we performed a multivariate Cox regression analysis limited to individuals without missing information in the fracture questionnaire for all years (*n* = 3,129) (Table 4). The results showed that the HRs of PTSD symptoms for individuals with fractures were similar to those presented in Table 2, indicating the robustness of the results in Table 2.

**Table 4:** Sensitivity analysis results of participants who responded to all surveys from 2011 to 2016

Factors	Classification	Adjusted HR (95% CI)^a^ (*n* = 3,129)	*P* value
Age	Continuous	1.05 (1.03–1.07)	< 0.001
Sex	Men	Ref	
	Women	2.09 (1.55–2.83)	< 0.001
PTSD symptoms	No	Ref	
	Yes	1.26 (0.99–1.59)	0.063
Experience of earthquake	No	Ref	
	Yes	0.99 (0.61–1.62)	0.982
History of mental illness	No	Ref	
	Yes	0.78 (0.47–1.31)	0.354
Need for assistance	No	Ref	
	Yes	0.89 (0.54–1.47)	0.642
History of cancer	No	Ref	
	Yes	1.71 (1.26–2.32)	< 0.001
History of stroke	No	Ref	
	Yes	1.34 (0.95–1.89)	0.098
History of heart disease	No	Ref	
	Yes	1.36 (1.07–1.73)	0.012
History of diabetes mellitus	No	Ref	
	Yes	1.37 (1.12–1.68)	0.003
Smoking habit	Never smoked	Ref	(trend *p* = 0.519)
	Former smoker	1.15 (0.85–1.55)	0.361
	Current smoker	1.07 (0.69–1.67)	0.751
Drinking habit	Never drinks or rarely drinks (less than once a month)	Ref	(trend *p* = 0.159)
	Former drinker	1.07 (0.63–1.81)	0.795
	Current drinker (more than once a month)	1.19 (0.93–1.53)	0.168
Level of sleep satisfaction	Satisfied with sleep	Ref	(trend *p* = 0.429)
	Slightly unsatisfied with sleep	1.07 (0.86–1.33)	0.557
	Quite unsatisfied with sleep	1.02 (0.73–1.41)	0.924
	Very dissatisfied with sleep or does not sleep at all	1.27 (0.79–2.02)	0.324
Exercise habit	Almost daily	Ref	(trend *p* = 0.919)
	2 to 4 times a week	1.12 (0.87–1.44)	0.374
	Approximately once a week	0.87 (0.62–1.21)	0.413
	Almost never	1.06 (0.80–1.39)	0.691

^a^Adjusted for age, sex, PCL score, experience of earthquake, history of mental illness, need for assistance, history of cancer, history of stroke, history of heart disease, history of diabetes mellitus, smoking habit, drinking habit, level of sleep satisfaction, and exercise habit. *95% CI* 95% confidence interval, *HR* hazard ratio, *Ref* reference, *PTSD* post–traumatic stress disorder. Cox proportional hazard model; *p* < 0.05 was considered statistically significant

## Discussion

Our study suggested that PTSD symptoms were significantly associated with the occurrence of fractures among older adults, particularly men, who resided in evacuation areas within Fukushima Prefecture. Studies have reported an increase in the prevalence of diseases, such as obesity and lifestyle-related diseases, in the residents of evacuation areas within Fukushima Prefecture [14–17]. This increase in disease prevalence could be partially attributed to the increase in stress caused by environmental changes due to moving into temporary housing, living in an evacuation site outside the local area, or disturbance in eating habits [18–21]. Thus, psychological stress has been considered to be associated with adverse health effects among residents of evacuation areas throughout Fukushima Prefecture. A study found that individuals who reported to have experienced high levels of psychological stress were at increased risk for fractures caused by osteoporosis [22]. One possible mechanism underlying the association between stress and fracture risk is that psychological stress increases cortisol secretion via the hypothalamus–pituitary–adrenal system. Glucocorticoids induce bone loss and increase the risk of osteoporotic fractures [23, 24]. Individuals with PTSD symptoms can be considered to have had high psychological stress immediately after a disaster. Furthermore, studies have reported that older adults and those living under extreme conditions were more likely to experience worse symptoms [25]. The psychological effects caused by the Fukushima nuclear accident have been widespread, causing not only trauma symptoms but also chronic and more complex social problems, such as stigma and community and family fragmentation [26]. Therefore, persistent high levels of stress caused by disasters could contribute to increased fracture risk in older adults. To prevent fractures after a disaster, older adults with PTSD symptoms should be assessed for bone mineral density and receive aggressive interventions to reduce psychosocial stress.

Furthermore, those with PTSD are presumed to have an increased likelihood of suffering from other mental disorders, such as depression [27, 28]. In previous studies, the percentage of residents with PCL scores above the cut-off point was significantly higher in residents with Kessler psychological distress scale (K6) scores [29–31] above the cut-off than in those with K6 score below the cut-off [32]. Furthermore, prefectural health surveys have reported that the coexistence of PTSD and previous mental illness or mental disorders were poor predictors of mid-term mental health [33]. Thus, the mental health deterioration caused by a disaster can promote even more confined and sedentary lifestyles among older adults who already tend to have low physical function in a depressed state, causing a decrease in physical function and a corresponding increased risk of fractures. Therefore, social participation should be encouraged in older adults with PTSD symptoms and low physical function to maintain and improve their physical function and mental health.

Depression itself has also been reported to be associated with an increased risk of fractures [34], which may be mediated by the use of antidepressants [35]. For instance, receiving one class of antidepressants, selective serotonin reuptake inhibitors (SSRIs), has been reported to increase the risk of fractures regardless of the presence of depression or bone density [36]. Moreover, SSRIs have been reported to contribute to fracture-induced falls and increased fracture risk [37]. Considering that SSRIs have occasionally been considered for the treatment of PTSD, older adults receiving medication for PTSD symptoms must be aware of the risk for fractures facilitated by antidepressants.

The present study found that those who were extremely dissatisfied with their sleep, particularly older men, were at increased risk of fractures. The prevalence of insomnia and use of sleeping pills among Japanese individuals have been reported to increase with age [38]. Benzodiazepines or benzodiazepine receptor agonists, a nonbenzodiazepine alternative, have been among the commonly prescribed sleeping pills in Japan. Accordingly, studies have shown that prolonged and high-dose usage of benzodiazepines was associated with an increased risk for falls and fractures [39–41], suggesting that insomnia pharmacotherapy could have also contributed toward increasing fracture risk among older adults, such as those residing in evacuation areas within Fukushima Prefecture. Understanding the sleep environment and providing guidance on sleep hygiene should be the initial management for insomnia. Our study suggests that securing sleep time and improving sleep quality are imperative for preventing fractures among older adults, particularly men, residing in evacuation areas within Fukushima Prefecture.

Our study found that women have a higher risk for fractures than men. However, although PTSD symptoms tended to be associated with the occurrence of fractures in women, it was not statistically significant. Moreover, sleep satisfaction was not significantly associated with the occurrence of fractures in women. In women, with regard to fractures, other factors may be more influential than the prevalence of PTSD symptoms and stress from lack of sleep. Primary osteoporosis among women is often caused by heredity, aging, and postmenopausal decline in female hormones [42]. Furthermore, patients with osteoporosis have been reported to be more likely to experience fractures after a fall [43]. Osteoporosis-related fractures can also have a significant impact on health-related QOL (HRQOL) [44]. Thus, the aforementioned results suggest that health problems that are specific to women, which could not be investigated herein, could have had a greater effect on fracture risk than the effect of increased psychological stress. However, exercise can be effective in reducing falls and risk factors associated with fractures from falls among patients with low bone mineral density [45]. Therefore, especially for women, regular bone density measurements and exercise habit formation for those with PTSD symptoms are recommended to prevent fractures from falling and a decline in HRQOL.

This study revealed that individuals who were current smokers and those with a history of diabetes, heart disease, stroke, and cancer were at an increased risk for fractures. Indeed, previous studies have reported that the prevalence of smoking habits [46], type 2 diabetes [47], cardiovascular disease [48], stroke [49], and cancer [50, 51, 52] increased the risk for fractures, suggesting that a comprehensive strategy, including smoking cessation to prevent lifestyle-related diseases, cardiovascular events, and cancer, is necessary for preventing fractures among older adults residents of evacuation areas.

The present study has certain noteworthy limitations. First, the age-adjusted prevalence of post-traumatic stress has been known to decrease every year, whereas studies have shown that the mental health of residents in evacuation areas within Fukushima Prefecture has improved compared to that at the time of the earthquake [53]. However, whether this improvement is prevalent among residents of the 13 municipalities remains unclear given that our participants comprised only a small percentage of those who participated in the Fukushima Health Management Survey. Horikoshi et al. also reported that those who did not respond to the mental survey had a significantly higher rate of psychological distress than the respondents [54]. Therefore, the results of this study could have underestimated the impact of increased PTSD symptoms caused by the Great East Japan Earthquake and FDNPS accident on fractures. Accordingly, it may be necessary to expand the scope of psychological research by including a survey on the mental health of nonrespondents.

Second, this survey did not include details on the medication conditions, bone density tests, fracture sites, circumstances during which fractures sustained, presence of osteoporosis, or use of antidepressants. Hence, factors that could contribute to fracture risk, such as the prevalence of osteoporosis and use of antidepressants and steroids [55], could not be investigated. Moreover, the effects of sex differences on fractures could not be completely clarified given that information on menopause or hormone levels among women was not surveyed. Therefore, future studies should include examinations and questions addressing these factors.

Third, studies on postmenopausal women have reported that obesity and underweight were both risk factors for fractures [56]. However, given that the present survey did not include items on height and weight in FY2011, body mass index could not be calculated. Therefore, we plan to examine the relationship between weight and fractures by evaluating health checkup data in our next study.

Fourth, the results of a systematic review and meta-analysis showed that frailty and pre-frailty were significant predictors of fractures among community-dwelling older adults [57]. Frailty can be assessed using the frailty index [58], which combines several variables (functioning, cognition, comorbidities, health attitudes and habits, and physical performance). However, the Mental Health and Lifestyle Survey does not include many questions on physical functioning, whereas the present survey items did not include the association between frailty and fractures. Future investigations may need to include a questionnaire on health examination results to screen for frailty.

Fifth, certain nutrients and foods have been reported to be associated with fracture risk [59, 60]. Previous studies on the Fukushima Health Management Survey also reported an association between psychological distress and food intake [20]. However, the Fukushima Health Management Survey contained a limited number of questions on food intake. Moreover, a clear bias was noted when evaluating each food group. Accordingly, we determined that data obtained from this study did not allow a comprehensive examination of the association between fractures and food intake and unfortunately, this information was not considered.

## Conclusions

The present study indicated that disaster-induced PTSD symptoms and insomnia contribute to increased fracture risk among older adults residing in evacuation areas within Fukushima Prefecture. Offering active psychological care to reduce psychosocial stress and providing guidance on sleep are important to prevent fractures in older adult residents, such as those living in evacuation areas.

## Data Availability

The datasets analyzed during the present study are not publicly available because the data of the Fukushima Health Management Survey belongs to the government of Fukushima Prefecture and can only be used within that organization.

## Abbreviations

PTSD: Post-traumatic stress disorder
PCL: Post-traumatic stress disorder checklist
HR: Hazard ratio
CI: Confidence interval
FDNPS: Fukushima Daiichi Nuclear Power Station
QOL: Quality of life
K6: Kessler psychological distress scale
SSRIs: Selective serotonin reuptake inhibitors
HRQOL: Health-related quality of life
FY: Fiscal year
SD: Standard deviation
Ref: Reference
